# Unique morphological architecture of the hamstring muscles and its functional relevance revealed by analysis of isolated muscle specimens and quantification of structural parameters

**DOI:** 10.1111/joa.13860

**Published:** 2023-03-13

**Authors:** Koichi Takeda, Kota Kato, Koichiro Ichimura, Tatsuo Sakai

**Affiliations:** ^1^ Department of Anatomy and Life Structure Juntendo University Graduate School of Medicine Tokyo Japan; ^2^ Department of Physical Therapy, Faculty of Health Science Juntendo University Tokyo Japan

**Keywords:** hamstrings, isolated muscle specimens, sarcomere length, structural parameters, superficial tendon

## Abstract

The structural and functional differences of individual hamstrings have not been sufficiently evaluated. This study aimed to clarify the morphological architecture of the hamstrings including the superficial tendons in detail using isolated muscle specimens, together with quantification of structural parameters of the muscle. Sixteen lower limbs of human cadavers were used in this study. The semimembranosus (SM), semitendinosus (ST), biceps femoris long head (BFlh), and biceps femoris short head (BFsh) were dissected from cadavers to prepare isolated muscle specimens. Structural parameters, including muscle volume, muscle length, fiber length, sarcomere length, pennation angle, and physiological cross‐sectional area (PCSA) were measured. In addition, the proximal and distal attachment areas of the muscle fibers were measured, and the proximal/distal area ratio was calculated. The SM, ST, and BFlh were spindle‐shaped with the superficial origin and insertion tendons on the muscle surface, and the BFsh was quadrate with direct attachment to the skeleton and BFlh tendon. The muscle architecture was pennate in the four muscles. The four hamstrings possessed either of two types of structural parameters, one with shorter fiber length and larger PCSA, as in the SM and BFlh, and the other with longer fiber length and smaller PCSA, as in the ST and BFsh. Sarcomere length was unique in each of the four hamstrings, and thus the fiber length was suitably normalized using the average sarcomere length for each, instead of uniform length of 2.7 μm. The proximal/distal area ratio was even in the SM, large in the ST, and small in the BFsh and BFlh. This study clarified that the superficial origin and insertion tendons are critical determinants of the unique internal structure and structural parameters representing the functional properties of the hamstring muscles.

## Graphical abstract

The three hamstring muscles (SM, ST, and BFlh) were equipped with membranous superficial tendons (epimuscular tendons) that were continuous with the tendons outside of the muscles (extramuscular tendons) at both the proximal and distal ends. The attachment areas of both the proximal and distal sides were arranged on opposite sides of the muscles, with the muscle belly in between. This positional relationship between the proximal and distal attachments has also been recognized in many other muscles and favors to maintain the fiber length within the muscle relatively constant.

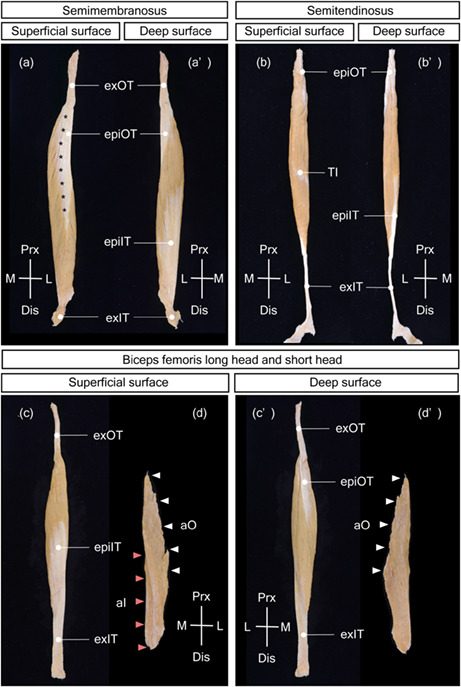

## 1 INTRODUCTION

The hamstrings are the muscle group in the posterior thigh that serves as the principal knee flexor and hip extensor. These muscles play an important role in activities of daily living, such as sit‐to‐stand (Hanawa et al., 2017), walking (Arnold et al., 2005; Winter & Yack, 1987), and sprinting (Chumanov et al., 2007; Thelen et al., 2005). The hamstrings are also a frequent site of muscle strain injury in various sports activities (Ekstrand et al., 2011; Volpi et al., 2004). For these reasons, the functional properties and status of hamstrings have been intensively studied in vivo by measuring muscle length (ML) with ultrasonography (Kellis et al., 2021) or recording muscle activity with electromyogram (Hirose et al., 2021).

The hamstrings consist of four muscles, namely, the semimembranosus (SM), semitendinosus (ST), biceps femoris long head (BFlh), and biceps femoris short head (BFsh), and collaborate in knee flexion and hip extension. Structural and functional differences in the four muscles have been suggested. The structural parameters vary among the four muscles (Charles et al., 2019; Kellis et al., 2012; Klein Horsman et al., 2007; Ward et al.,  2009; Wickiewicz et al., 1983), suggesting that they have significantly different functional characteristics. Muscle strains have been reported to be frequent both at the proximal muscle–tendon junction of the BFlh and in the proximal tendon of the SM, depending on the mode of motion (Askling et al., 2007a, 2007b).

The superficial origin and insertion tendons were reported as an integral part of muscle architecture in the hamstrings by Woodley and Mercer (2005), Kellis et al. (2010) and van der Made et al. (2015), and also in other fusiform muscles as reported by Sakai and Kato (2021). The tendons were recognized as dense connective tissue that connected the muscular tissue to the skeleton and may be designated as aponeurosis in the flattened form. They could be subdivided into the free tendon without muscular tissue (extramuscular part) and the surface tendon on the muscular tissue (epimuscular part). While the extramuscular tendons serve to transmit the whole muscle forces to the skeleton, the epimuscular tendons providing wide attachment areas to the muscle fibers are critical determinants of the functional and clinical characteristics of the muscles. Although the structural parameters and functional properties were reported to be different among the four hamstring muscles, the difference of the morphology of epimuscular tendons and the arrangement of muscle fibers have not been well elucidated in the previous studies.

It is well known that muscle tension is maximal at the optimal sarcomere length (SL) and decreases considerably with lengthening or shortening of the sarcomere (Lieber et al., 1994). On this basis, Lieber and Friden (2000) recommended normalizing the fiber length (FL) with the measured and optimal SL to calculate the physiological cross‐sectional area (PCSA). Burkholder and Lieber (2001) rationalized the normalization of FL in a review article by showing that the SLs reported in 29 articles were different among animal species as well as among individual muscles and among different methods and different researchers. Felder et al. (2005) measured both FL and SL in the mouse tibialis anterior at different joint angles and normalized the FL with high precision. SL has been reported to change with different joint angles in various muscles in humans (Chen et al., 2016; Chen & Delp, 2016; Cromie et al., 2013; Lichtwark et al., 2018; Ljung et al., 1999) and monkeys (Ando et al., 2021). In calculating the structural parameters of muscles, Lieber and Friden (2000) recommended normalizing the FL with the measured SL and a uniform optimal SL of 2.7 μm reported by Walker and Schrodt (1974). However, since different values of SL among the individual muscles were known from recent studies reporting the structural parameters (Borst et al., 2011; Cutts, 1988; Ward et al., 2009), the calculating methods of structural parameters should be re‐evaluated.

In the present study, we examined the internal structure of the hamstrings using isolated muscle specimens to clarify the morphology of the epimuscular tendons and the internal muscle architecture, and in addition to evaluate structural parameters critically as a sound basis of functional properties of the hamstrings.

## 2 METHODS

### 2.1 Source of cadavers

We used cadavers of persons who had donated their bodies for medical education and research to Juntendo University School of Medicine. Before donation, written consent from donors and their families was obtained.

Sixteen left legs were collected from formaldehyde‐embalmed Japanese body donors (8 males 82.0 ± 6.4 years old, and 8 females 88.4 ± 6.4 years old), which were dissected by medical students in the gross anatomy course at Juntendo University School of Medicine. We do not have information on weight and height of the individual cadavers in this study. In our dissection protocol for medical students, the right legs were dissected to observe the bones and ligaments; thus, the muscles, nerves, and vasculature were more or less completely destroyed. Therefore, we only used the hamstrings of the left leg, where there was no serious structural damage to the muscles. We excluded cadavers that exhibited significant pathological alterations in muscles (such as muscular dystrophy, fatty degeneration, and large intramuscular hematomas), traumatic lesions, surgical scars, and flexion contracture of the knee joint. Each cadaver was formaldehyde‐embalmed in an anatomical position with negligible flexion of the joints (less than 10 degrees). Photographs were reversed to represent the right side.

### 2.2 Preparation and observation of isolated hamstrings

The skin, subcutaneous tissue, superficial and deep fasciae, gluteus maximus, and neurovascular bundles were removed from the posterior thigh to reveal the hamstring muscles. The SM, ST, and BFlh were then released from the ischial tuberosity, and the BFsh was removed from the lateral lips of the femur. Subsequently, the insertion of the SM, ST, BFlh, and BFsh was removed from the medial condyle of the tibia, tibial tuberosity, and fibular head to prepare isolated whole hamstrings. The whole hamstring was then divided into individual SM, ST, BFlh, and BFsh muscles.

### 2.3 Measurement

Structural parameters were measured in isolated specimens of individual muscles. The ML and FL were measured using a tape measure. The pennation angle (PA) was measured using a protractor. The ML were measured as distances between the proximal and distal ends of the muscle body containing muscle fibers, excluding the extramusclar tendons.

The FL and PA were measured along the fascicles beginning from 9 points plotted evenly on both sides of the edge from top to bottom (5 points on one side in the BFsh) of the proximal or distal superficial tendon on the muscle surface. The PA was measured on the muscle surface as the angle between the individual fascicles and the long axis of the muscle.

The mean values and standard deviations of each FL and PA were subsequently calculated using SPSS.

The SL was measured under a light microscope (Olympus, BX53F) at five points in each of the five muscle fibers obtained from each of the 16 individual specimens for each of the SM, ST, BFlh, and BFsh. Optimal SL was calculated using the following formula (Lieber et al., 1994):

$$\mathrm{Lf}={\mathrm{Lf}}^{’}\times \mathrm{Ls}/{\mathrm{Ls}}^{’}$$

where Lf is the optimal fiber length, Lf' is the raw fiber length, Ls is the standard sarcomere length, and Ls' is the raw sarcomere length.

The standard SL was defined as the average of the measured SLs for the individual muscles.

The PCSA was estimated using the following equation (Lieber & Friden, 2000; Powell et al., 1984):

$$\text{PCSA}\;\left({\mathrm{cm}}^{2}\right)=M\left(g\right)\times \text{cosθ}/\rho \;\left(g/{\mathrm{cm}}^{3}\right)\times \mathrm{Lf}\;\left(\mathrm{cm}\right)$$

where θ is the pennation angle and ρ is the muscle density (1.056 g/cm^3^) (Klein Horsman et al., 2007; Ward & Lieber, 2005).

The average cross‐sectional area (AvCSA) was estimated using the following equation:

$$\text{AvCSA}\;\left({\mathrm{cm}}^{2}\right)=\text{Muscle Volume}\;\left({\mathrm{cm}}^{3}\right)/\mathrm{ML}\;\left(\mathrm{cm}\right)$$

The proximal and distal attachment areas of the fascicles to the tendon or skeleton were measured on the photographs of isolated muscle specimens using Adobe Photoshop. The muscle fiber attachment areas were defined as those areas on the skeleton or tendinous tissue on which the muscle fibers attach, namely the areas of epimuscular tendons in SM and BFlh, the areas of epimuscular tendons plus the attachment areas on the BFlh proximal tendon in ST, and attachment areas on the lateral lips of the femur and on the BFls distal tendon. The muscle fiber attachment area ratio was calculated from dividing the attachment areas by the total muscle areas measured on the photographs.

### 2.4 Statistical analyses

The muscle fiber attachment area ratio was compared using a paired t‐test.

SLs and normalized PCSA were compared between groups using analysis of variance followed by the Bonferroni test as a post hoc test; *P* values of <0.05 were considered statistically significant. The PCSA of the four hamstrings were compared among three groups after normalization with different standard values of sarcomere length. (group 1, Ls = average; group 2, Ls = 2.7 μm; group 3, without normalization). All analyses were performed using SPSS software.

## 3 RESULTS

### 3.1 Anatomy of hamstrings in situ

**FIGURE 1:**
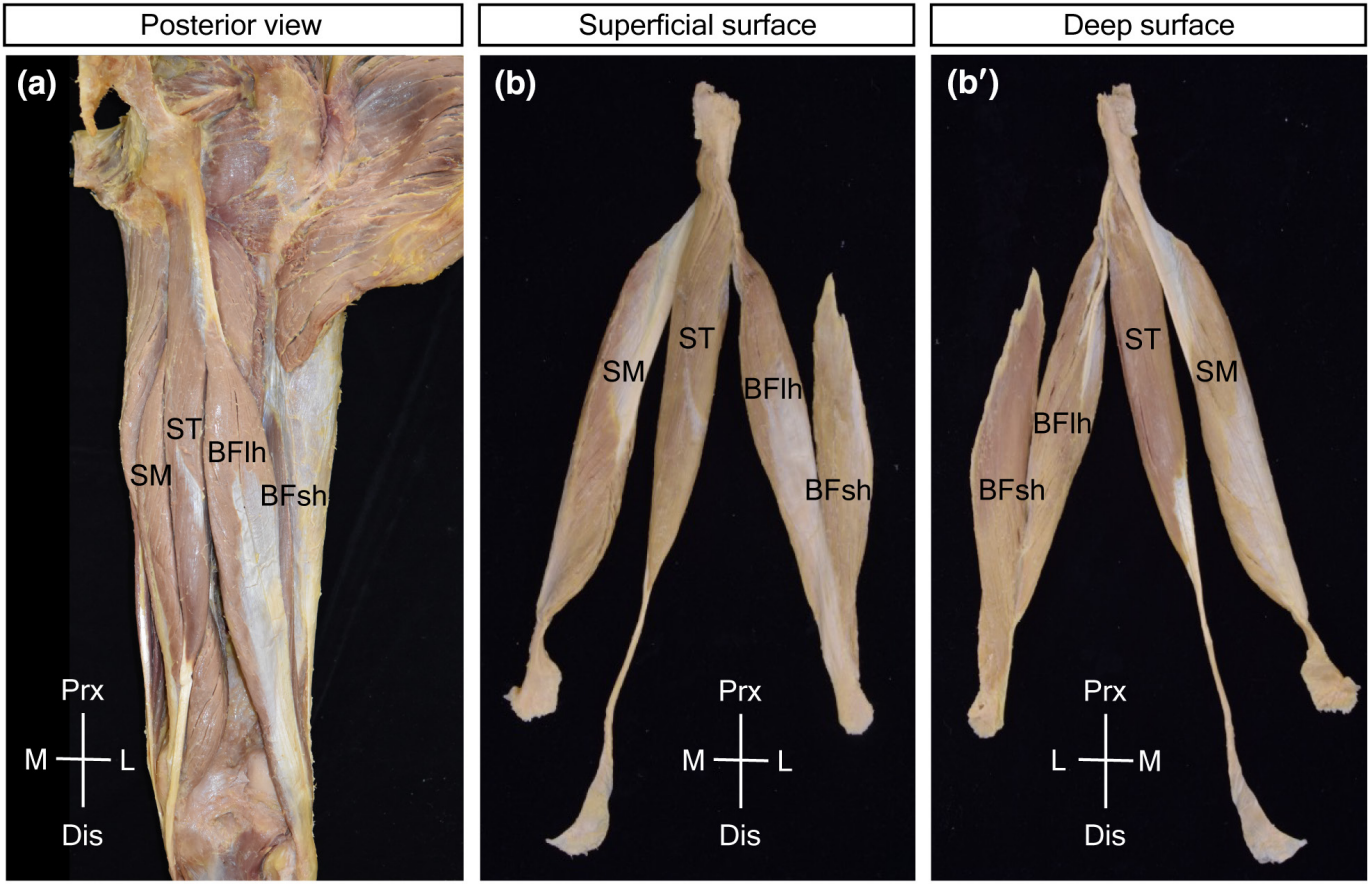
The four hamstrings in situ and in isolated specimens. (a) In situ posterior view of the hamstrings in the posterior thigh. (b, b’) Isolated specimens of the whole hamstrings in the superficial surface (b) and deep surface (b’). BFlh, biceps femoris long head; BFsh, biceps femoris short head; SM, semimembranosus; ST, semitendinosus.

With in situ observation, the origin and insertion tendons of the hamstrings were only partially visible. The origin tendons of the BFlh and ST were superficially located and visible, whereas those of the SM were deeply located and were completely concealed. The superficial insertion tendon of the BFlh on the posterior surface of the muscle was clearly visible, and a similar superficial insertion tendon of the SM was only partially visible on the lateral edge of the muscle (Figure 1a). It was obvious that evaluation of the superficial origin and insertion tendons on the muscular surface was only possible after isolation of the hamstrings.

By observing the isolated specimens (Figure 1b,b'), the superficial origin and insertion tendons of the SM, BFlh, and ST were fully uncovered. The BFlh possessed a superficial origin tendon on the deep surface and a superficial insertion tendon on the superficial surface of the muscle. The SM possessed a superficial origin tendon on the superficial lateral surface and a superficial insertion tendon on the deep medial surface. For the ST, the superficial insertion tendon was poorly developed and found on the deep medial surface of the muscle. The present observations revealed that the origin and insertion tendons outside the muscle (extramuscular tendon) continued frequently on the muscles as membranous superficial tendons (epimuscular tendon). Furthermore, it became evident that the SM and BFlh were equipped with well‐developed epimuscular origin and insertion tendons on the opposing surfaces of the muscles and that the ST possessed a poorly developed epimuscular insertion tendon on the opposite side of its origin attachment to the BFlh origin tendon.

The structure of the epimuscular origin and insertion tendons and the arrangement of the muscular fascicles were analyzed in isolated specimens of the individual muscles.

### 3.2 Architecture of the hamstring muscles

#### 3.2.1 Semimembranosus

**FIGURE 2:**
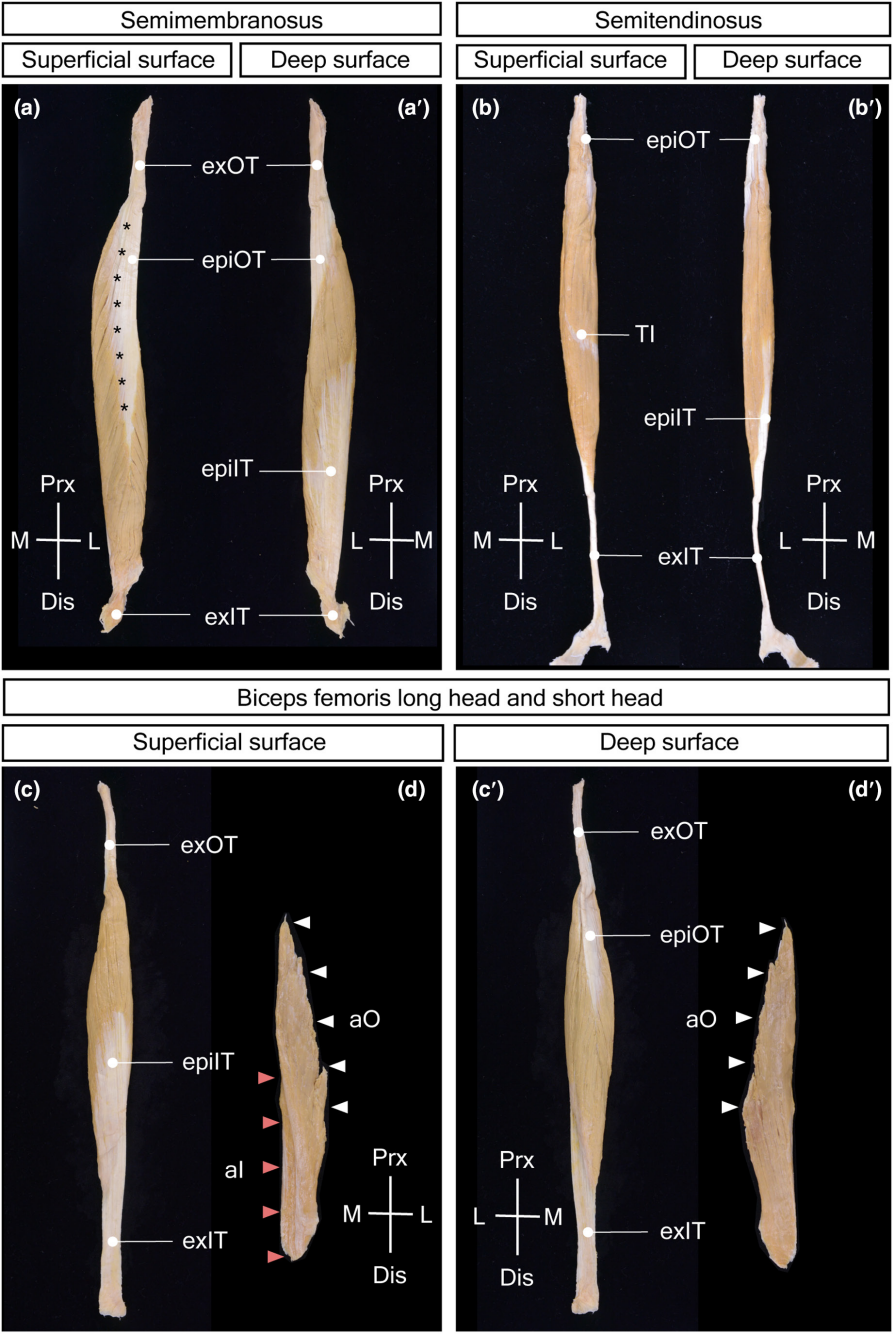
Isolated specimens of the SM, ST, BFlh, and BFsh. (a, a’) SM in the superficial surface (a) and deep surface (a'). The slender extramuscular origin tendon (exOT) outside of the muscle continued to the membranous epimuscular origin tendon (epiOT) on the superficial lateral surface of the muscle. The membranous epimuscular insertion tendon (epiIT) spreads widely on the deep medial surface of the muscle and continues to the short extramuscular insertion tendon (exIT). The epimuscular origin tendon is subdivided into a thicker axial part and a thinner wing part at the boundary indicated by asterisks. (b, b') ST in the superficial surface (b) and deep surface (b'). The origin tendon of ST possesses no extramuscular part and extends shortly as the epimuscular origin tendon (epiOT) on the lateral surface of the muscle. The epimuscular insertion tendon (epiIT) on the medial surface of the muscle continued to a long string‐like extramuscular insertion tendon (exIT). (c, c’) BFlh in the superficial surface (c) and deep surface (c'). The slender extramuscular origin tendon (exOT) continues to the narrow band‐like epimuscular origin tendon (epiOT) on the deep surface. The epimuscular insertion tendon (epiIT) covers the superficial surface of the muscle and continues to the strap‐shaped extramuscular insertion tendon (exOT). (d, d') BFsh in the superficial surface (d) and deep surface (d'). The edge‐like proximal end of BFsh possesses no tendon attaching muscularly to the femur (aO); white arrowheads—attachment site of origin side. The thicker distal end of BFsh also possesses no tendon attaching to the deep surface on the extramuscular insertion tendon of BFlh (aI); red arrowheads—attachment site of insertion side. aO, attachment site of origin side; aI, attachment site of insertion side; epiIT, epimuscular insertion tendon; epiOT, epimuscular origin tendon; exIT, extramuscular insertion tendon; exOT, extramuscular origin tendon.

The origin tendon of the SM consisted of the slender and thick extramuscular tendon and the membranous epimuscular tendon on the superficial lateral surface extending down to the distal third of the muscle (Figure 2a). The epimuscular tendon possessed a composite substructure with a thicker axial part and thinner wing part (indicated by asterisks in Figure 2a). The axial part was composed of parallel bundles of tendon fibers, which continued at the tip of the epimuscular tendon as radiating branches within the muscle (intramuscular tendon). The wing part spread away from the axial part as a thin sheet and ran obliquely parallel to the muscle fibers.

The insertion tendon of the SM consisted of a significantly short stout extramuscular tendon and membranous epimuscular tendon on the deep medial surface, surrounding the distal half up to the middle of the muscle (Figure 2(a)’). The epimuscular tendon was homogeneous in structure and radiated from the distal end, becoming thinner toward the proximal end.

**FIGURE 3:**
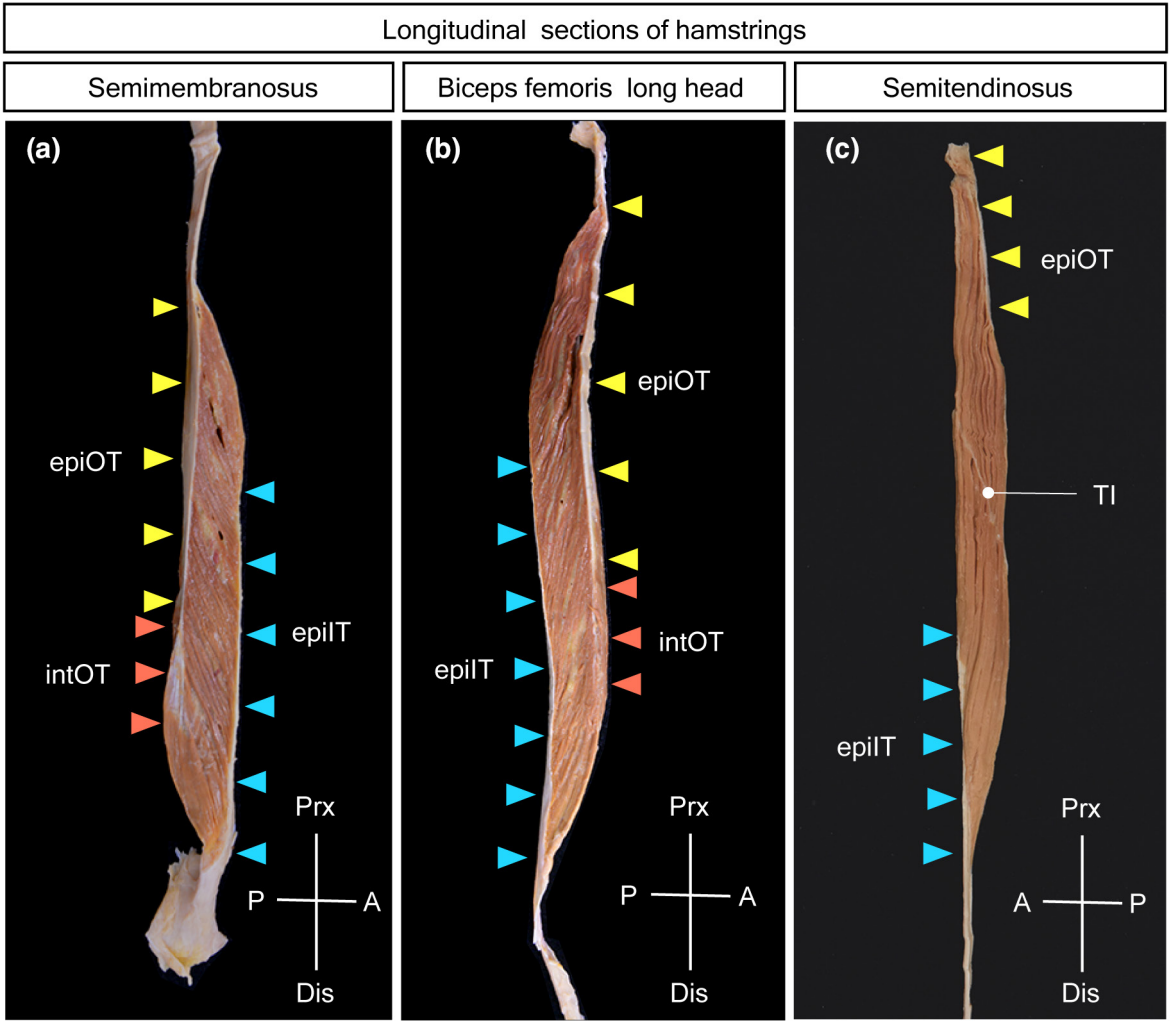
Longitudinal sections of the hamstrings through both the proximal and distal epimuscular tendons. (a) In the SM, the muscle fibers connect obliquely between the epimuscular origin tendon (epiOT) and epimuscular insertion tendon (epiIT) on the opposite sides, exhibiting pennate architecture with short muscle fibers. The tip of epimuscular origin tendon continues as radiating branches within the muscle (IntOT, intramuscular origin tendon). (b) In the BFlh, the muscle fibers connect obliquely between the epimuscular origin tendon (epiOT) and epimuscular insertion tendon (epiIT) on the opposite sides, showing pennate architecture with short muscle fibers. The epimuscular tendon becomes thinner and more slender distally and slips into the muscle to become an intramuscular tendon (IntOT). (c) In the ST, the muscle fibers connect diagonally between the proximal and distal attachment sites at the muscle extremities (epiOT, epiIT), presenting pennate architecture with relatively long muscle fibers. The muscle fibers of ST are interrupted by a disc‐like tendinous inscription (TI) in the middle of the muscle. epiIT, epimuscular insertion tendon; blue arrowheads—area of epiIT; epiOT, epimuscular origin tendon; yellow arrowheads—area of epiOT; IntOT, intramuscular origin tendon; brown arrowheads—area of IntOT; TI, tendinous inscription.

The membranous epimuscular tendon at the origin and insertion sides was located on the opposite side of the muscle and spread equally in the area. The muscular bundles connected both epimuscular tendons and were arranged oblique to the longitudinal axis of the muscle, so that the muscle had a parallel pennate architecture (Figure 3a). The FL was significantly short in comparison with the muscle body length, as was evident in the small value of the muscle fiber ratio (approximately 20%) in this muscle.

#### 3.2.2 Semitendinosus

The ST did not form a specific origin tendon outside of the muscle and attached to the medial part of the ischial tuberosity via a small amount of tendinous connective tissue at the proximal end and to the medial surface of the origin tendon of the BFlh below it. At the attachment to the BFlh tendon, the ST adhered to the BFlh origin tendon muscularly in the superficial part and through the superficial origin tendon of the ST in the deep part. The superficial origin tendon extended to the upper third of the muscle (Figure 2b,b').

The insertion tendon of the ST consisted of a long string‐like extramuscular tendon and a narrow epimuscular tendon on the deep medial side of the muscle. The epimuscular tendon extended to the distal third of the muscle. The muscle fiber connected the origin structure on the upper end and lateral surface with the epimuscular insertion tendon and ran longitudinally almost parallel to the muscle axis (Figure 2b').

The muscle fibers of the ST were interrupted by a disc‐like tendinous inscription in the middle of the muscle (Figure 2b). The tendinous disc was inclined approximately 70° (69.1°°± 4.17°) with respect to the cross‐section of the muscle, with the proximal end on the medial and distal ends on the lateral surface of the muscle. The disc was slightly convex on the upper side so that the border of the disc found on the muscle surface was widened near the lower end and narrowed in the other parts. The upper end of the tendinous inscription was frequently covered by muscle fibers, which passed through the uninterrupted space between the origin and insertion structures. The muscle fibers were arranged diagonally between the origin and insertion structures, but tended to run parallel along the muscle length in the middle, especially near the tendinous inscription.

The origin structure of the ST was located on the upper lateral part, and the insertion epimuscular tendon was found on the lower medial side of the muscle so that the musculotendinous connection areas on the proximal and distal sides were placed in parallel on the opposite side of the muscle (Figure 3c). The origin area was twice as wide as the insertion area, and the muscle fibers converged from the origin side to the insertion side, resulting in a converging pennate muscle architecture. The tinting and convexity of the tendinous interruption corresponded to the arrangement of the origin structures and insertion epimuscular tendon. The muscle fibers of the ST were longer than the muscle body length (approximately 54%) in this muscle.

#### 3.2.3 Biceps femoris long head

The origin tendon of the BFlh consisted of the slender extramuscular tendon and the narrow band‐like epimuscular tendon on the deep medial surface extending down to the center of the muscle (Figure 2c'). The epimuscular tendon became thinner and more distally slender and slipped into the muscle to become an intramuscular tendon down to the distal third of the muscle.

The insertion tendon of the BFlh possessed a strap‐shaped extramuscular tendon and a broad epimuscular tendon covering the superficial lateral surface and extending up to the middle of the muscle body (Figure 2c). The epimuscular tendon was homogeneous in structure and radiated from the distal end becoming thinner toward the proximal end, similar to that of the SM.

The origin and insertion epimuscular tendons were located on the opposite surfaces of the muscle, and their areas on the muscle were quite different, being much smaller on the origin side than on the insertion side. The muscular bundles extended from the narrow origin epimuscular tendon obliquely to the muscle axis and radially to the broad insertion epimuscular tendon so that the muscle had a radiating pennate architecture (Figure 3b). The FL was significantly short in comparison with the muscle body length, as was evident from the small value of the muscle fiber ratio (approximately 28%) in this muscle.

#### 3.2.4 Biceps femoris short head

The BFsh did not form separate origin and insertion tendons but attached muscularly to the linea aspera of the femur on the proximal side and to the extramuscular insertion tendon of the BFlh on the distal side (Figure 2d,d'). The proximal border of the muscle was thin and elongated vertically with a slender attachment area at the lateral edge of the muscle. The muscle became thicker and narrower distally and was attached to the deep surface of the insertion tendon of the BFlh. The BFsh had a parallel pennate architecture. The muscle fibers of the BFsh were not significantly short compared with the muscle body length (approximately 45%).

### 3.3 Structural parameters of the hamstring muscles

**FIGURE 4:**
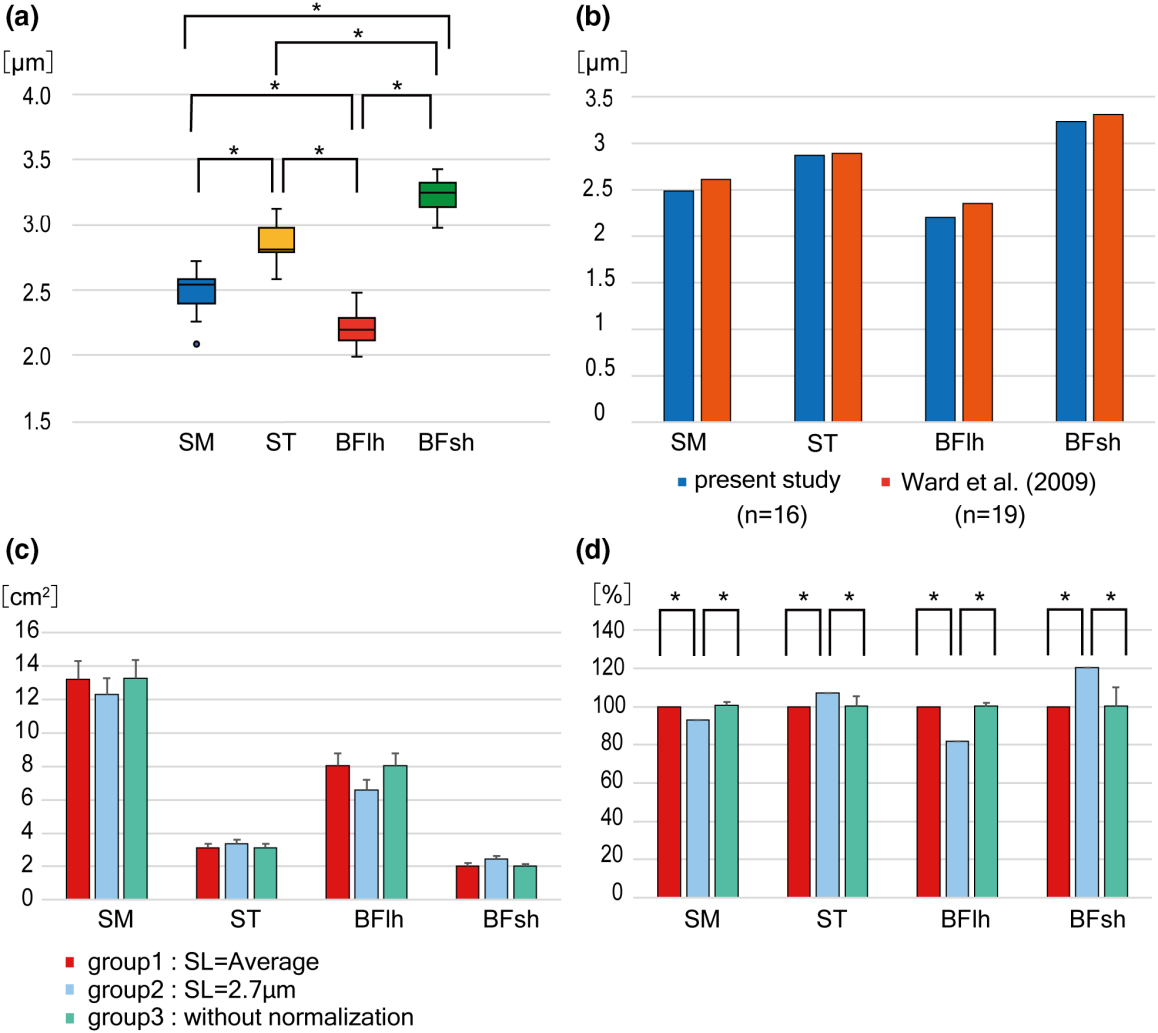
Normalization of PCSA with the different sarcomere lengths. (a) Average values of the sarcomere lengths in the individual hamstrings. The significant differences between values (*p* < 0.05) are indicated by asterisks. (b) Average values of sarcomere lengths of the individual hamstring muscles in the present study and Ward et al. (2009). (c, d) Normalized PCSA of the four hamstrings with different standard values of sarcomere length (bar 1, Ls = average; bar 2, Ls = 2.7 μm; bar 3, without normalization) shown as absolute values (c) and as relative values (100% = average value of sarcomere length) (d). The values are significantly different (*p* < 0.05) between groups 1 and 2 as well as groups 2 and 3 in the relative values for each of the muscles. BFlh, biceps femoris long head; BFsh, biceps femoris short head; SM, semimembranosus; ST, semitendinosus.

The SM, ST, and BFlh were spindle‐like, and the BFsh was a quadrate; all of them were categorized as pennate in architecture. However, they displayed manifest structural differences in the expansion of the epimuscular tendons and extent of the muscle–tendon junctions, which must be correlated with the functional properties of the muscles. To evaluate the functional properties of these muscles, we measured the size and length of the muscles and fibers to calculate the PCSA, AvCSA, FL‐to‐ML (FL/ML) ratio, and PCSA‐to‐AvCSA (PCSA/AvCSA) ratio (Table 1). The average value of the SL was significantly different among the muscles (Figure 4a). The normalized PCSA values were compared with different standard values of SL for each of the four hamstrings in absolute values (Figure 4c) and in relative values (Figure 4d). The values are significantly different between groups 1 and 2 as well as groups 2 and 3 in the relative values for each of the muscles. Thus, in the present study, ML and FL were normalized using the average SL of 16 individuals instead of 2.7 μm.

**TABLE 1:** Descriptive structural parameters of the hamstring muscles.

Muscle	Muscle volume (cm^3^)	ML (cm)	FL (cm)	SL (μm)	PA(°)	PCSA (cm^2^)	AvCSA (cm^2^)	FL/ML ratio	PCSA/AvCSA ratio
SM (*n* = 16)	71.26 ± 27.22 (38.20%)	25.59 ± 2.86 (11.19%)	5.15 ± 0.53 (10.23%)	2.42 ± 0.14 (6.43%)	13.82 ± 2.56 (18.56%)	13.22 ± 4.18 (31.63%)	2.77 ± 0.98 (35.47%)	0.20 ± 0.03 (13.36%)	4.78 ± 0.72 (14.78%)
ST (*n* = 16)	50.60 ± 17.03 (33.66%)	29.88 ± 2.65 (8.86%)	15.96 ± 1.20 (7.54%)	2.86 ± 0.11 (4.82%)	9.20 ± 2.18 (23.64%)	3.14 ± 1.02 (32.45%)	1.69 ± 0.51 (30.28%)	0.54 ± 0.04 (7.11%)	1.85 ± 0.12 (6.65%)
BFlh (*n* = 16)	57.23 ± 20.94 (36.58%)	25.39 ± 2.29 (9.03%)	7.01 ± 0.71 (10.08%)	2.20 ± 0.13 (6.11%)	10.31 ± 2.26 (21.90%)	8.04 ± 2.86 (35.50%)	2.26 ± 0.80 (35.42%)	0.28 ± 0.03 (9.71%)	3.58 ± 0.36 (10.02%)
BFsh (*n* = 16)	26.81 ± 8.27 (30.84%)	27.39 ± 2.06 (7.54%)	12.33 ± 1.11 (9.04%)	3.23 ± 0.13 (4.02%)	19.16 ± 3.29 (17.17%)	2.04 ± 0.60 (29.22%)	0.98 ± 0.31 (31.56%)	0.45 ± 0.03 (6.76%)	2.10 ± 0.18 (8.54%)

*Note*: Values are expressed as mean ± standard deviation and coefficient of variation.

Abbreviations: AvCSA, average cross‐sectional area; BFlh, biceps femoris long head; BFsh, biceps femoris short head; FL, fiber length; ML, muscle length; PA, pennation angle; PCSA, physiological cross‐sectional area; SL, sarcomere length; SM, semimembranosus; ST, semitendinosus.

The muscle volume was maximum in the SM, followed by the BFlh and ST, and the BFsh was small. However, individual differences were extremely large, as shown by the coefficient of variation (CV) (30.8%–38.2%).

The difference in ML among the four muscles was small. Individual differences were small, as shown by the CV (7.5%–11.2%).

**FIGURE 5:**
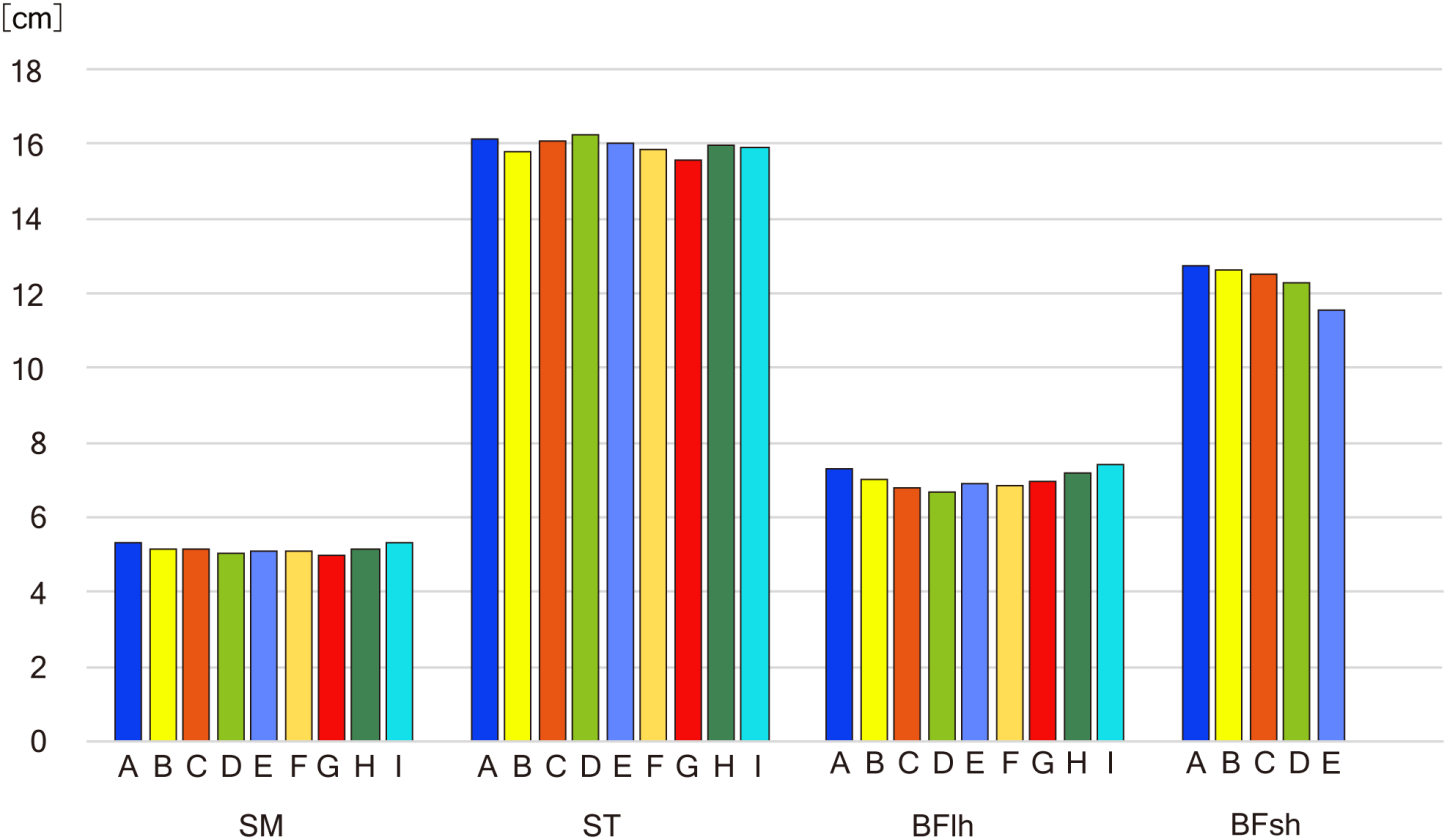
Intramuscular variation of FL was measured at nine points in SM, ST, and BFlh, and five points in BFsh. The central point (the tip of origin tendon in SM and BFlh, the tip of insertion tendon in ST) or the most distal point in BFsh was indicated by E. BFlh, biceps femoris long head; BFsh, biceps femoris short head; SM, semimembranosus; ST, semitendinosus.

The FLs of the SM (5.15 cm) and BFlh (7.01 cm) were relatively short, and those of the ST (15.96 cm) and BFsh (12.33 cm) were relatively long. Individual differences were small, as shown by the CV (7.5%–10.2%). The difference of FL was found within individual muscles and was obviously much smaller than the difference among the four hamstring muscles (Figure 5). The distal fascicles arising around the tip of origin tendon were shorter than the proximal fascicles by 7.0% in SM and 10.9% in BFlh, and the distal fascicles in BFsh were shorter than the proximal ones by 9.9%.

The SL was the longest in the order of the BFsh (3.23), ST (2.86), SM (2.42), and BFlh (2.20). Individual differences were small, as shown by the CV (4.0%–6.4%).

The PA showed almost no difference in the PA between the measurement sites in the SM (13.82°) and ST (9.20°). In the BFlh (10.31°), the outer side of the proximal tendon tended to have a larger PA than the inner side. In the BFsh (19.16°), the PA tended to be large distally and smaller toward the proximal side. Individual differences were large, as shown by the CV (17.2%–23.6%).

For PCSA, the SM (13.22 cm^2^) was the largest, followed by the BFlh (8.04 cm^2^), and the ST (3.14 cm^2^) and BFsh (2.04 cm^2^) tended to be smaller. Individual differences were extremely large, as shown by the CV (29.2%–35.5%).

The AvCSAs of the SM, BFlh, ST, and BFsh were 2.77, 2.26, 1.69, and 0.98 cm^2^, respectively, showing the same tendency as the size of PCSA of the four muscles. Individual differences were extremely large, as shown by the CV (30.3%–35.5%).

The FL/ML ratio was small in the SM (0.20) and BFlh (0.28) and large in the BFsh (0.45) and ST (0.54). Individual differences were small, as shown by the CV (6.8%–13.4%).

The PCSA/AvCSA ratios of the SM, BFlh, BFsh, and ST were 4.78, 3.58, 2.10, and 1.85, respectively, showing the same tendency as the size of PCSA of the four muscles. Individual differences were small, as shown by the CV (6.7%–14.8%).

The coefficients of variation for the FL/ML and PCSA/AvCSA ratios were small and stable.

### 3.4 The ratio of the proximal and distal attachment areas of muscle fascicles to the tendon or skeleton

**FIGURE 6:**
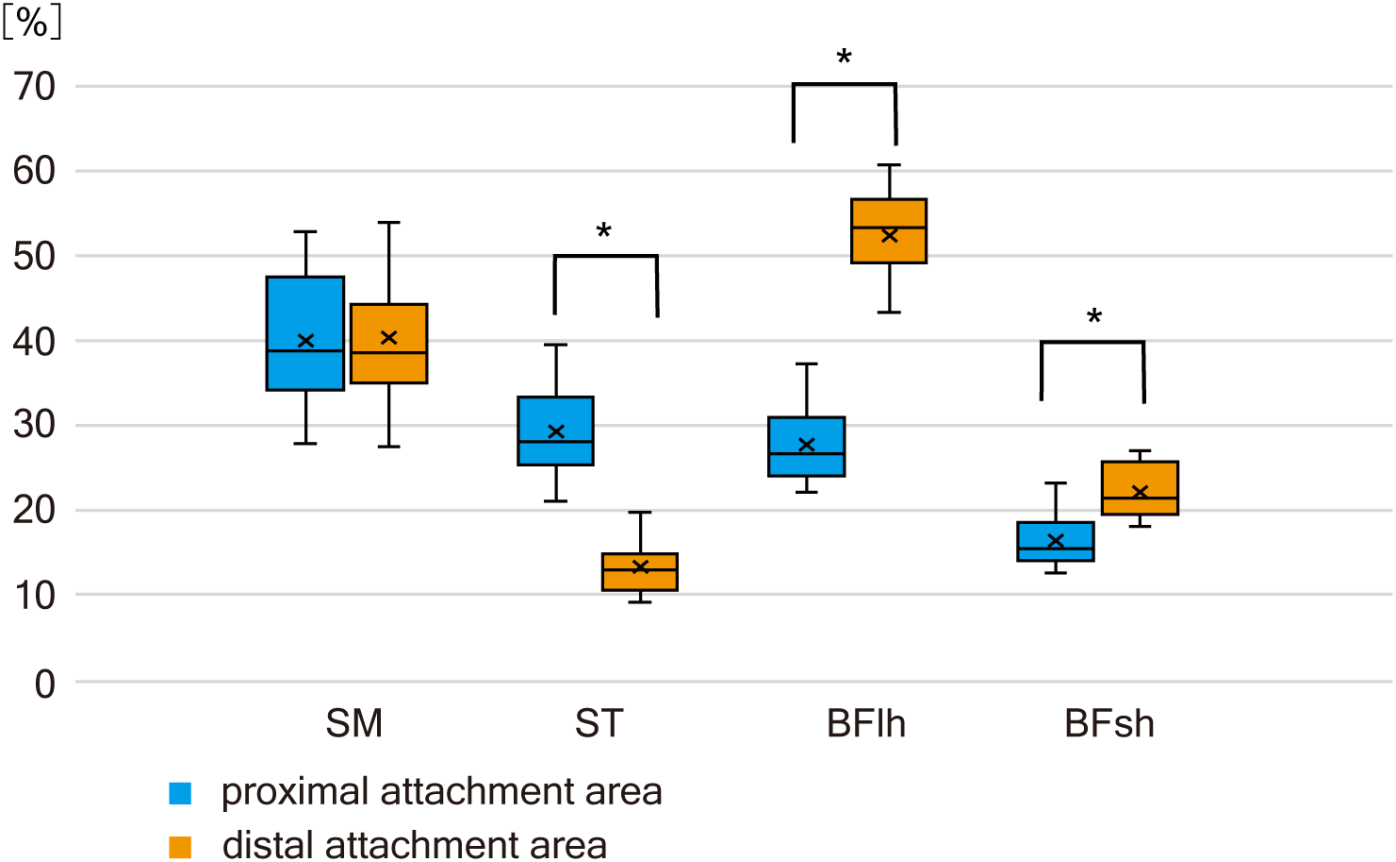
The ratio of the proximal and distal attachment areas of muscle fibers on the epimuscular tendons or extramuscular skeletal structures to the whole muscle areas evaluated on the photographs of isolated muscle specimens. Differences of the area ratio to the muscle surface are tested using a pair *t*‐test. *p < 0.05. BFlh, biceps femoris long head; BFsh, biceps femoris short head; SM, semimembranosus; ST, semitendinosus.

In the present study, we observed that the individual hamstring muscles frequently possessed superficial proximal and distal tendons, on which the muscle fascicles attached, in addition to the attachment to the extramuscular skeleton and tendinous structures. The size and shape of the superficial tendons were diverse, as exemplified by the narrowness of the superficial proximal tendon and expansion of the superficial distal tendon in the long head of the biceps femoris. Therefore, the area of attachment of the muscle fascicles was measured on the proximal and distal sides of the individual muscles (Figure 6).

The proportion of attachment area to the total muscle area was calculated for each muscle. The average proportion values for the proximal and distal attachments were almost equal in the SM (40.18%) and BFlh (40.09%) and smaller in the ST (21.25%) and BFsh (19.25%). In addition, the ratio of proportion values between the proximal and distal attachments was different among the muscles, being balanced in the SM (0.99) and unbalanced in the BFlh (0.53). In the ST (2.20), the proportion of the proximal attachment was larger than that of the distal attachment, and in the BFsh (0.74), the proximal proportion was slightly smaller.

We conducted a paired t‐test for the proportion of proximal and distal attachment areas and found a significant difference between the proximal and distal attachment areas in the ST, BFlh, and BFsh.

## 4 DISCUSSION

### 4.1 Muscle architecture and shape of hamstrings

We clarified the morphology of the attachment areas of the muscle fibers in the hamstrings. The attachment of the muscle fibers was provided by the epimuscular tendons in the SM, ST, and BFlh and by the extramuscular skeletal and tendinous elements in the BFsh. The attachment areas of both the proximal and distal sides were arranged on opposite sides of the muscle, with the muscle belly in between. This positional relationship between the proximal and distal attachments has also been recognized in many other muscles and can be called the principle of opposition of the origin and insertion surfaces (Sakai & Kato, 2021).

Kellis et al. (2010) and Woodley and Mercer (2005) studied the variation of FL in the individual hamstring muscles, and we confirmed that the differences of FL between the muscles surpassed far those within the muscles (Figure 5), as reported in the dorsal muscle of the scapula (Langenderfer et al., 2006) and in the flexor muscle of the forearm (Liu et al., 2014). The opposing origin and insertion epimuscular tendons or structures are favored to maintain the FLs relatively constant (Sakai & Kato, 2021).

The muscle shape was fusiform in the SM, ST, and BFlh and quadrate in the BFsh, and the muscle architecture was pennate in all four muscles. In previous anatomy textbooks, the architecture of fusiform muscles differed from that of pennate muscles, and their muscle fibers were arranged in longitudinal directions (Moore, 1980; Romanes, 1972; Rosse & Gaddum, 1997; Schaeffer, 1953; Williams, 1995). As shown in the present study, the concept of “fusiform” concerned solely the shape of the muscle and not the architecture of the muscle, whereas the concept of “pennate” concerned solely the architecture of the muscle and not the shape of the muscle.

### 4.2 Normalization of FL by SL

In the normalization of FL, the optimal SL was usually assumed to be 2.72 μm based on the study by Walker and Schrodt (1974) who measured the SL in several muscles using transmission electron microscopy without verifying the difference in SL among the individual muscles. In fact, the SLs were considerably different among muscles, as reported by Cutts (1988), showing variation in SL in 10 lower limb muscles between 1.970 and 3.007 μm. Thereafter, variations in SL among muscles were frequently shown in studies to calculate the PCSA, as 2.66–3.07 μm in the three muscles posterior to the scapula (Langenderfer et al., 2006), 2.12–3.31 μm in 28 muscles of the lower limbs (Ward et al., 2009), 2.52–2.77 μm in 3 muscles of the pelvic floor (Alperin et al., 2014), 2.16–2.78 μm in 5 muscles of the forearm (Liu et al., 2014), 1.98–3.64 μm in 35 cervical muscles (Borst et al., 2011), 2.14–3.57 μm in 11 trunk muscles (Bayoglu et al., 2017), and 2.45–2.85 μm in 8 hip muscles (Parvaresh et al., 2019). These studies strongly indicated the possibility that the optimal SLs differed among the muscles. Furthermore, recent studies have shown that SL is longer in cases of cerebral palsy (Lieber & Friden, 2019; Mathewson et al., 2014, 2015; Smith et al., 2011) and that the SL and number decreased in contracted muscles during apoplexy (Adkins et al., 2021), indicating the probable SL change in adaptation to muscle activity. The currently available information does not support the assumption that the optimal SLs are single and constant among the different muscles in the human body.

In the present study, we obtained different average values of SL among the four hamstring muscles, which would provide similar change of the muscle fiber length during the flection and extension of the hip and knee joints.

The difference in the average SL among these muscles could not be explained by the different states of muscle contraction or fixation procedures. Furthermore, the average SLs of these four muscles were distinctly different and exhibited constant order of magnitude in every cadaver, namely, BFsh (average, 3.23 μm), ST (average, 2.86 μm), SM (average, 2.42 μm), and BFlh (average, 2.20 μm) from the largest (Figure 4a).

Ward et al. (2009) reported the corresponding difference and order of SLs in four hamstring muscles (Figure 4b). These results do not support the hypothesis of a single and constant value of optimal SL but indicate that the individual muscles possess unique different values of optimal SL. As the most suitable approximation of optimal SL for individual muscles, we employed the average SL among the individual cadavers instead of a constant value of 2.7 μm. Compared with the PCSA calculated with individually different optimal SLs, the PCSA values with a constant optimal SL of 2.7 μm were calculated to be 20% larger or smaller, indicating the possibility of inaccurate over‐ or underestimation (Figure 4c,d).

### 4.3 Relevance of quantitative measurements

The structural parameters were measured in three hamstrings to calculate the PCSA and other muscle parameters in the present study. Similar data reported in previous studies (Charles et al., 2019; Kellis et al., 2012; Ward et al., 2009) were too diverse to obtain standard values for individual muscles. The diversity of structural parameters was known to be correlated to some extent with the individual differences affected by age, sex, and race but possibly by the differences in the employed methods, such as direct measurement on the cadaveric specimens or virtual measurement on the MRI.

The degree of individual diversity was evaluated using the CV, obtained by dividing the standard deviation value by the average in the present study, indicating the relative variation of the measured values. The CV of the muscle parameters was quite large in the muscle volume (30.8%–38.2%) and much smaller in the ML (7.5%–11.2%) and FL (7.5%–10.2%). The large CV of the muscle volume may be reasonable because the volume is generally proportional to the third power of the length. The CV of body weight in Japanese children (13.7%–22.5%) was approximately four times larger than the CV of body height (3.4%–5.3%) by Annual Report of School Health Statistics Researches (2019). Furthermore, the anatomical cross‐sectional area of the thigh muscles was reported to increase by 9%–14% (Farup et al., 2012), suggesting considerable changes in muscle volume even in the same individuals, whereas the ML and FL remained constant. Thus, the muscle volume and PCSA, which are dependent on muscle volume, were thought to be highly variable among individuals or even in the same individual so that no standard values could be expected.

**FIGURE 7:**
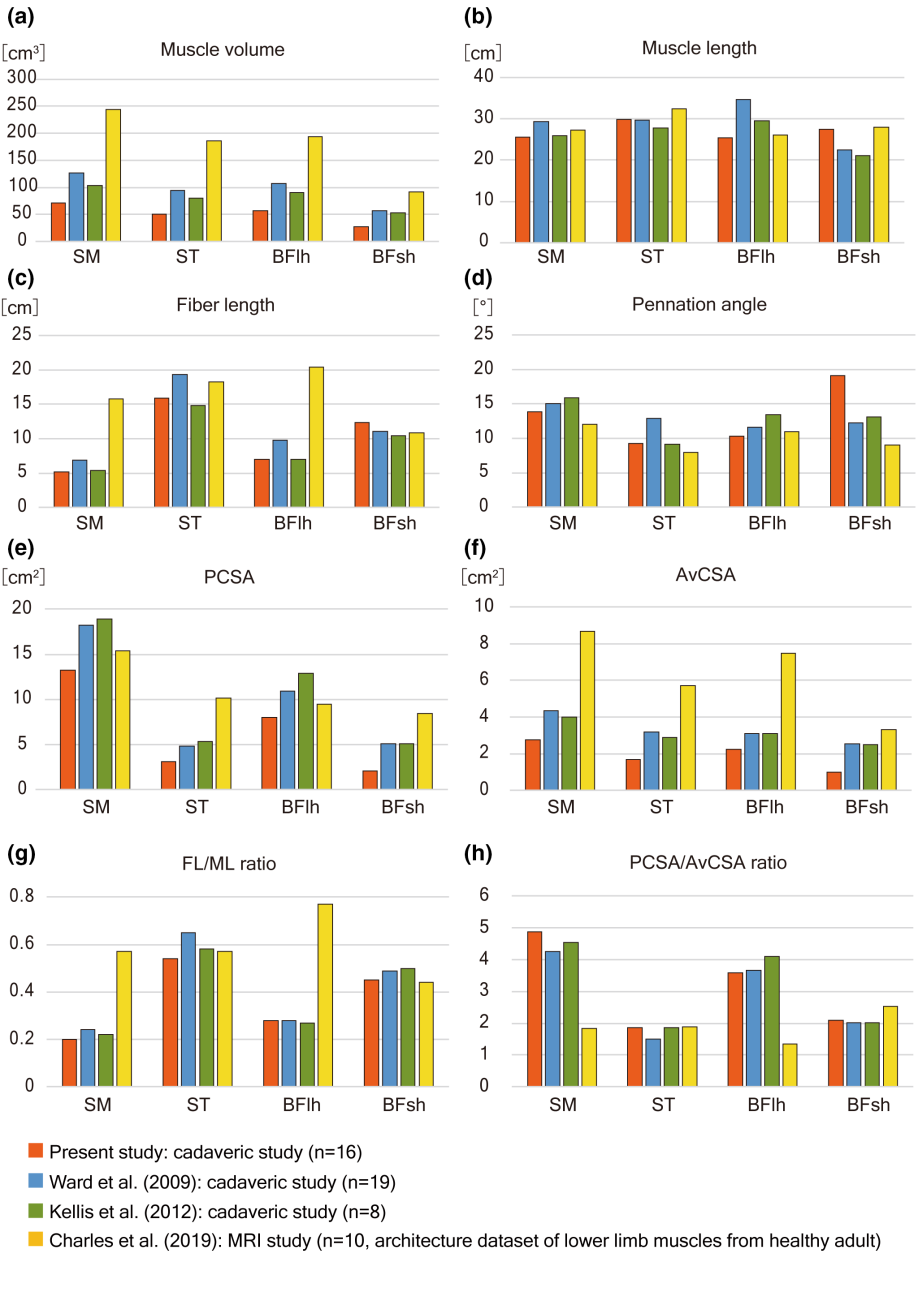
Structural parameters of the hamstring muscles as they appear in the literatures and in the present study. The graphs compare three cadaveric studies (Takeda, the present study; Kellis et al., 2012); Ward et al., 2009 and an MRI study (Charles et al., 2019). (a) Muscle volume, (b) Muscle length (ML), (c) Fiber length (FL), (d) Pennation angle, (e) physiological cross‐sectional area (PCSA), (f) average cross‐sectional area (AvCSA), (g) FL/ML ratio, and (h) PCSA/AvCSA ratio. BFlh, biceps femoris long head; BFsh, biceps femoris short head; SM, semimembranosus; ST, semitendinosus.

The structural parameters of hamstrings reported in previous and present studies employing cadaver specimens (Ward et al., 2009; Kellis et al., 2012; present study) or three‐dimensional (3D) images reconstructed from MRI (Charles et al., 2019) were variable to some extent. The considerable differences in the muscle volumes between the three cadaveric studies (SM, 71–127 cm^3^; ST, 51–94 cm^3^; BFlh, 57–107 cm^3^; BFsh, 27–57 cm^3^) and the MRI study (SM, 244 cm^3^; ST, 186 cm^3^; BFlh, 194 cm^3^; BFsh, 92 cm^3^) could be easily explained as individual differences, including age differences (Figure 7a). The MLs of the four muscles and FLs in the ST and BFsh were almost homogeneous among the four studies (SM, 25.6–29.3 cm; ST, 27.7–32.4 cm; BFlh, 25.4–34.7 cm; BFsh, 21.2–27.9 cm) (Figure 7b). On the other hand, the FLs in the SM (5.2–6.9 cm) and BFlh (7.0–9.8 cm) according to the three cadaveric studies were relatively small and homogeneous, whereas those according to the MRI study were more than twice as large (SM, 15.8 cm; BFlh, 20.4 cm) (Figure 7c). Thus, the FL/ML ratio reported by Charles et al. (2019) was more than 0.5 (SM, 0.57; BFlh, 0.77) and exceedingly larger than expected from the characteristics of pennate muscle with short muscle fibers, as revealed by the present and previous anatomical studies (Figure 7g). When the 3D reconstructed images by Charles et al. (2019) were examined, the superficial proximal and distal tendons were not distinguished from the muscle fibers and were included in the range of measurement of the FL. This fact indicates that the exceptionally large FLs of SM and BFlh in the study by Charles et al. (2019) resulted from the misidentification of muscle fibers to be measured and not from the differences between cadaveric and living tissues, as they suggested.

The muscle volume and PCSA were significantly variable among individuals, as indicated by the large coefficient variation (30.8%–38.2% for muscle volume and 29.2%–35.5% for PCSA) compared with the ML and FL (7.5%–11.2% for ML and 7.5%–10.2% for FL). The PCSA would be inappropriate as a general index for the functional characteristics of individual muscles but useful as a specific index for the degree of development and muscle strength in individuals. Muscle hypertrophy after exercise is known to result from the enlargement of individual muscle fibers (Farup et al., 2012). The PCSA/AvCSA ratio calculated in this study as PCSA divided by AvCSA was theoretically 1.0 in the perfect fusiform muscle with FL equal to ML, and much larger in the pennate muscle with FL shorter than ML, and would be useful as a general index of the functional characteristics of individual muscles. The PCSA/AvCSA ratio in the hamstring muscles was relatively constant among individuals (Figure 7h), as indicated by the much smaller CV (6.7%–14.8%) than PCSA (29.2%–35.5%), and expected to be a specific value for the functional properties of the muscle as FL/ML.

### 4.4 Functional characteristics of individual hamstrings predicted by the architecture

Muscle strain in the hamstrings is most frequent in the BFlh, especially at the proximal muscle–tendon junction (Askling et al., 2007a). Recently, Fiorentino and Blemker (2014) and Rehorn and Blemker (2010) reported a narrow superficial origin tendon on the muscle surface of the BFlh with MRI, and deduced susceptibility for muscle strain at the proximal muscle‐tendon junction on the basis of a 3D mechanical model and finite element analysis. In the present study, with anatomical analysis of isolated muscle specimens, we confirmed that the muscle‐tendon junction area at the proximal side of the BFlh was smaller compared with the distal side and the other hamstrings quantitatively after morphometry. If we may reasonably premise that the every muscle fiber span between the proximal and distal attachment, it would be logically concluded that the concentration of muscle fibers at the proximal side of the BFlh exceeds far the distal side of BFlh and the other hamstrings, a condition indicating susceptibility for muscle strain at this muscle‐tendon junction.

## AUTHOR CONTRIBUTIONS

Conceptualization, KT and TS; cadaver preparation, KI; data curation, KT, TS, and KK; data validation, KT and TS; and manuscript writing, KT and TS.

## CONFLICT OF INTEREST STATEMENT

The authors declare no potential conflict of interest.

## Data Availability

On reasonable request, the obtained data supporting the finding of the present study are available from the corresponding authors.
